# Technologies and applications of single-cell DNA methylation sequencing

**DOI:** 10.7150/thno.82582

**Published:** 2023-04-23

**Authors:** Fang Liu, Yunfei Wang, Hongcang Gu, Xiaoxue Wang

**Affiliations:** 1 Anhui Province Key Laboratory of Medical Physics and Technology, Institute of Health and Medical Technology, Hefei Institutes of Physical Science, Chinese Academy of Sciences, Hefei, 230031, China.; 2 University of Science and Technology of China, Hefei, 230026, China.; 3 Hefei Cancer Hospital, Chinese Academy of Sciences, Hefei, 230031, China.; 4 Zhejiang ShengTing Biotech. Ltd, Hangzhou, 310000, China.; 5 Department of Hematology, the First Hospital of China Medical University, Shenyang, 110001, China.

**Keywords:** DNA methylation, single-cell sequencing, single-cell multi-omics sequencing

## Abstract

DNA methylation is the most stable epigenetic modification. In mammals, it usually occurs at the cytosine of CpG dinucleotides. DNA methylation is essential for many physiological and pathological processes. Aberrant DNA methylation has been observed in human diseases, particularly cancer. Notably, conventional DNA methylation profiling technologies require a large amount of DNA, often from a heterogeneous cell population, and provide an average methylation level of many cells. It is often not realistic to collect sufficient numbers of cells, such as rare cells and circulating tumor cells in peripheral blood, for bulk sequencing assays. It is therefore essential to develop sequencing technologies that can accurately profile DNA methylation using small numbers of cells or even single cells. Excitingly, many single-cell DNA methylation sequencing and single-cell omics sequencing technologies have been developed, and applications of these methods have greatly expanded our understanding of the molecular mechanism of DNA methylation. Here, we summaries single-cell DNA methylation and multi-omics sequencing methods, delineate their applications in biomedical sciences, discuss technical challenges, and present our perspective on future research directions.

## Introduction

DNA methylation refers to the phenomenon in which a methyl group (CH3) from S-adenosylmethionine is transferred to the C-5 position of cytosine by DNA methyltransferases (DNMTs) 1, 2. DNA methylation is the most stable epigenetic modification. Another type of DNA methylation in mammals occurs at the N-6 position of adenine, although its functions are still under extensive investigation 3, 4. The 5-methylcytosine (5mC) is the dominant type of DNA modification, accounting for approximately 1% of the human genome 5. It occurs almost exclusively in the form of 5'-3' cytosine-phosphate-guanine (CpG) dinucleotides, and approximately 70-80% of CpGs are methylated in mammals 6, 7. CpGs are not randomly distributed across the genome, but exhibit widely scattered and locally clustered distributions 8. The CpG-rich regions, where the C+G content exceeds 50% and the observed to expected CpG ratio is equal to or greater than 0.6, are called CpG islands (CGIs). CGIs are typically 300-3,000 bp in length and overlap with 60% of human gene promoters and almost 100% of housekeeping gene promoters 9-12. Like CGIs, most CpG-rich regions show low levels of methylation, whereas CpG-poor regions are generally hypermethylated in mammals 13, 14. Remarkably, gene regulatory elements, including enhancers and transcription-factor binding sites, exhibit dynamic DNA methylation across tissues and cell types 15.

DNA methylation plays a critical role at the molecular, biological, and pathological levels 6, 13, 14, 16. Promoter hypermethylation is often associated with gene silencing and has been frequently observed in cancer 17. The repressive role of 5mC at gene promoters can be caused by directly preventing transcription factors (TFs) from binding to the corresponding elements, thereby blocking gene transcription. Alternatively, 5mC attracts methyl-CpG-binding domain (MBD) proteins to attach to promoter regions, consequently blocking TF binding to regulatory elements 14, 18. However, DNA methylation in the gene bodies shows complicated correlations with transcription: most low and highly-expressed genes exhibit low levels of methylation in the gene bodies, whereas moderately expressed genes show the highest levels of methylation 19. DNA methylation is also associated with increased levels of C-to-T mutations 8, 15, 20. Repetitive elements, which comprise more than 55% of the human genome, consist mainly of retrotransposons and are the primary targets of DNA methylation 21. Loss of DNA methylation in repetitive elements contributes to genome instability and global hypomethylation, which are considered hallmarks of cancer 14, 16, 17, 22, 23. Notably, the mammalian genome undergoes two waves of global demethylation and remethylation during development. In the first wave, primordial germ cells undergo genome-wide DNA demethylation, forming identical hermaphroditic epigenomes and ultimately developing new sex-specific epigenetic modifications 24, 25. The second wave occurs immediately after fertilization, initially erasing the methylation profiles of the gametes and later rewriting the methylation markers of the embryos 26. Mammalian development is also characterized by X chromosome inactivation (XCI) and genomic imprinting, resulting in monoallelic gene expression 27-29.

The biochemical processes of methylation and demethylation are beginning to be elucidated. *De novo* methylation is catalyzed by DNMT3A, 3B, and 3L, while the methylation profiles of dividing cells are maintained by DNMT1 26. On the other hand, demethylation is the result of an active enzymatic process and passive replication-dependent dilution. Specifically, 5mC is first converted to 5*-*hydroxymethylcytosine (5hmC), then to 5-formylcytosine (5fC), and finally to 5-carboxylcytosine (5caC) under the catalysis of ten-eleven translocation enzymes (TET1, 2, 3) 30-33. These oxidized derivatives of 5mC are diluted during DNA replication. Alternatively, 5fC and 5caC are actively removed by thymine DNA glycosylase (TDG) and subsequently replaced by cytosine through base excision repair (BER) 34. The dynamic nature of DNA methylation is attracting scientists to develop epigenetic drugs. Among them, DNA methyltransferase inhibitors, 5-azacytidine (Azacytidine, Vidaza®) and 5-aza-2'-deoxycytidine (Decitabine, Dacogen®), are the most successful epigenetic drugs. These drugs have been approved by the FDA and are widely used to treat patients with myelodysplastic syndrome (MDS) 35, 36.

DNA methylation analysis is essential to dissect its role in the development and human diseases, such as cancer. Methods for DNA methylation detection, including the principles and applications, have been described in the literature 8, 37-42. Next-generation sequencing (NGS)-based assays combined with the sodium bisulfite treatment are widely adopted due to their high reproducibility and accuracy at the single base level 37. In particular, whole-genome bisulfite sequencing (WGBS) is considered as the gold standard; however, the high sequencing costs and a significant amount of non-CpG reads make it less efficient for DNA methylation detection 8, 38. Reduced representation bisulfite sequencing (RRBS) enriches CpG-rich fragments using restriction endonucleases, such as MspI (C|CGG) and HaeIII (GG|CC), and gel-based size selection. It therefore significantly reduces sequencing costs while still covering most CpG islands and promoters, a good representation of other genomic features, including enhancers and CpG island shores 43-45. Conventional NGS-based methods, such as WGBS and RRBS, require a large amount of DNA, mainly measure the average DNA methylation level of many cells, and cannot identify the methylation status of individual cells 37. Yet, cellular heterogeneity is a pervasive phenomenon in multicellular organisms, suggesting that the NGS-based measurements may not reflect the actual methylation status. The accessibility of certain rare cells, such as embryonic stem cells in early development and cancer stem cells, also limits the application of bulk sequencing methods 46. Therefore, DNA methylation profiling at the single-cell level is essential, and many such technologies have been reported recently 47.

In this review, we summarize single-cell sequencing methods for the assessment of DNA methylation alone or in combination with other omics, outline the applications, and present with our perspective on these technologies.

## Single-cell isolation

**Figure 1:**
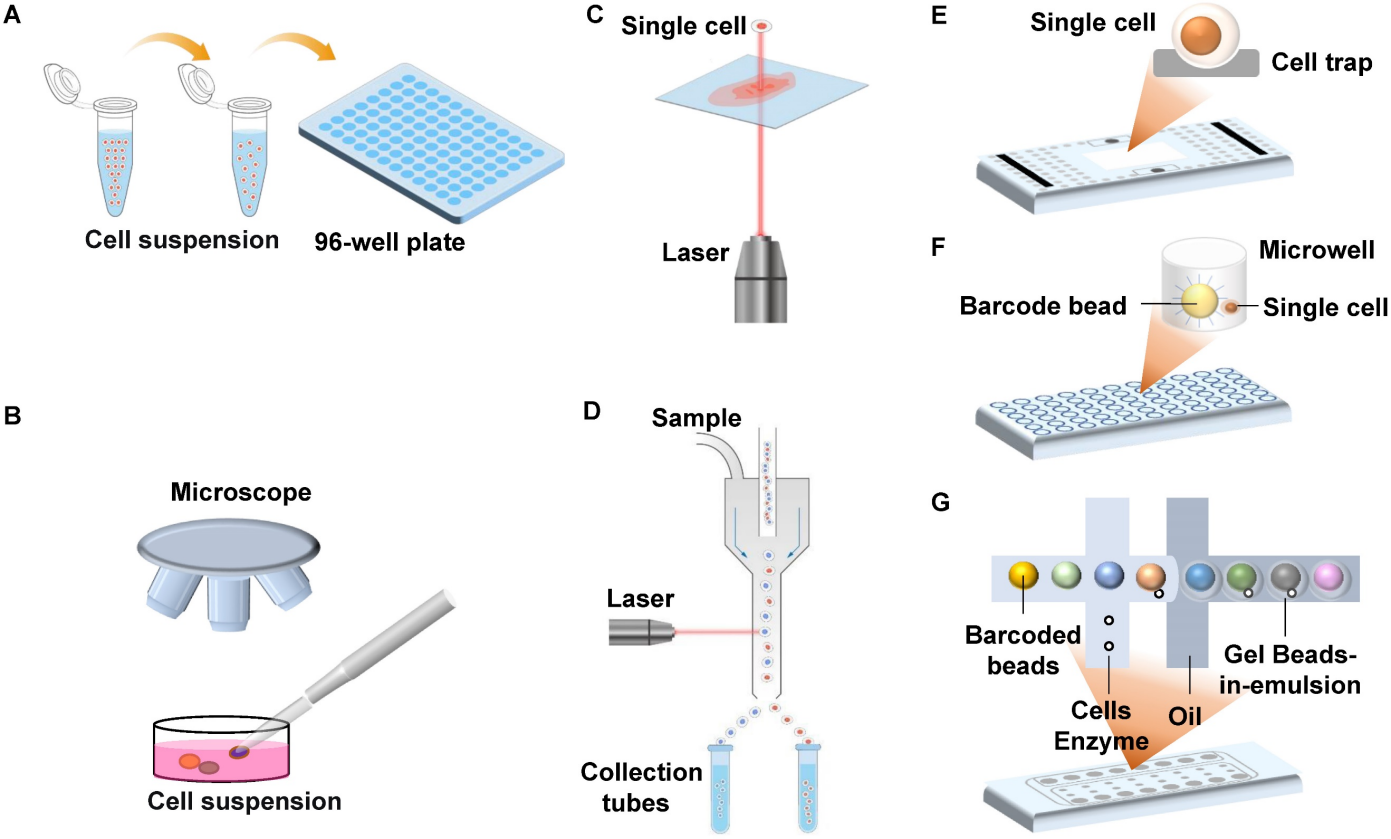
Schematic of single-cell isolation technologies. (A) Random limiting dilution; (B) Micromanipulation; (C) Laser capture microdissection; (D) Fluorescence activated cell sorting; (E) Microfluidic devices based on hydrodynamic cell traps; (F) Microwell-based microfluidics; (G) Droplet-based microfluidics.

Isolating intact individual cells is crucial for single-cell sequencing, and various methods have been documented and summarized in **Table 1** 48. The limiting dilution method is characterized by low cost and low throughput (**Figure 1A**). Micromanipulators utilize automated pipetting under microscopic observation, allowing operators to isolate single cells efficiently and accurately (**Figure 1B**). Laser capture microdissection (LCM) also allows targeted cell collection under microscopic visualization. The difference is that LCM focuses on isolating single cells from stained tissue and is, therefore, able to collect single cells with specific histological characteristics (**Figure 1C**).

Many single-cell isolation platforms use flow cytometry or microfluidic devices to automatically sort single cells with high throughput. The most common platform is the fluorescence-activated cell sorting (FACS) system (**Figure 1D**), and one of its main advantages is the throughput, which enables the isolation of hundreds of single cells within 30 minutes 49. Secondly, the platform can sort cells according to their functional properties using fluorescent staining, thus targeting individual cells of interest 50. Most commercial single-cell sorting platforms are based on microfluidic technology, such as the C1™ Single-Cell Auto Prep System (**Figure 1E**), the BD Rhapsody™ Single-Cell Analysis System (**Figure 1F**), and the 10× Chromium Single Cell Gene Expression Solution (**Figure 1G**) 51, 52. Commercial platforms can simultaneously perform cell sorting and barcode each cell, improving sequencing throughput and reducing sequencing costs.

## Single-cell DNA methylation sequencing

**Figure 2:**
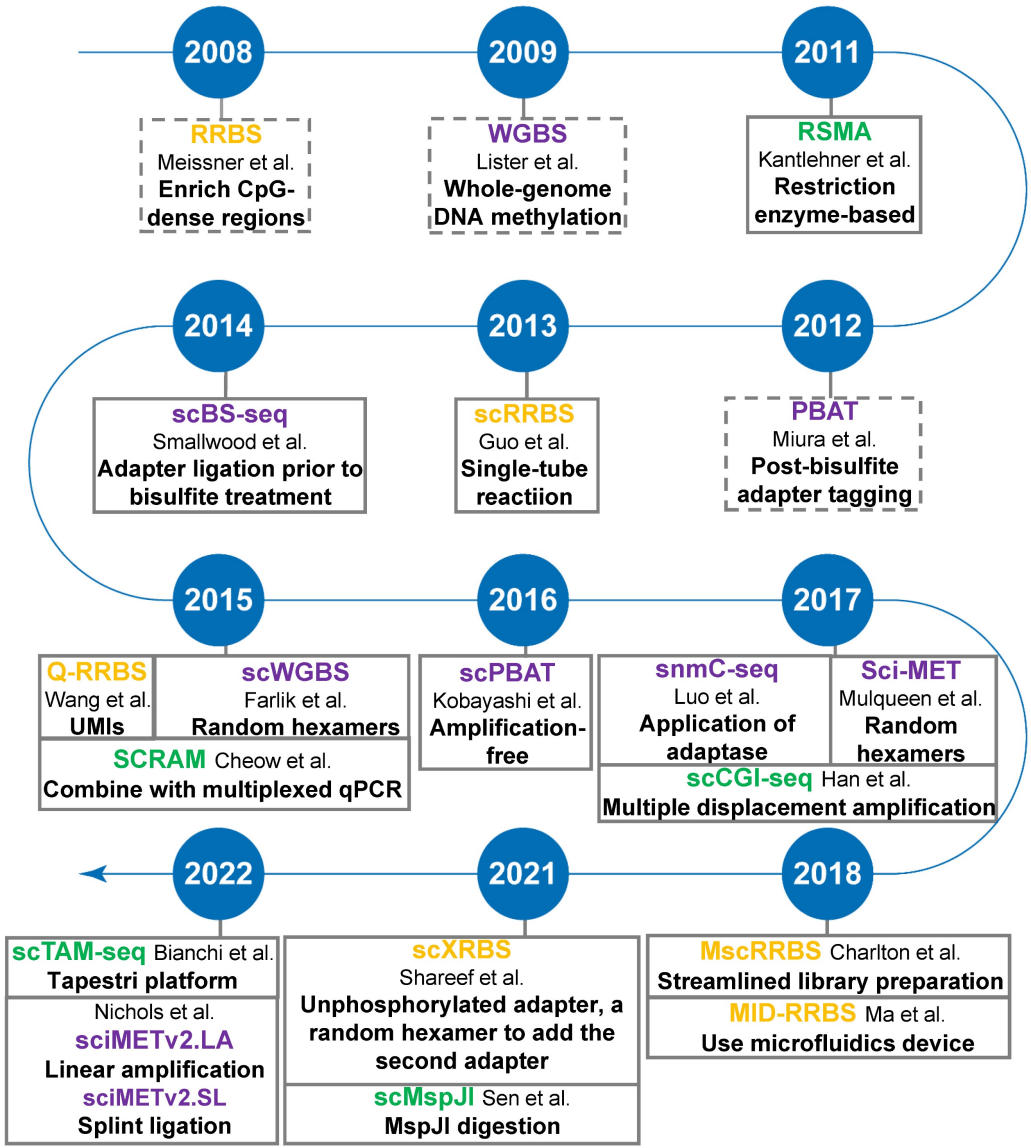
Timeline of single-cell DNA methylation sequencing technologies. Yellow-highlighting: RRBS-based single-cell DNA methylation methods; Green-highlighting: MSRE-based single-cell DNA methylation methods; Purple-highlighting: PBAT-based single-cell DNA methylation methods. Dotted line square: conventional bulk sequencing assays, including RRBS, WGBS, and PBAT.

The advent of single-cell isolation technologies and the optimization of methylation sequencing technologies are accelerating the development of single-cell DNA methylation sequencing. Most, if not all, single-cell sequencing methods are based on the corresponding bulk-based assays. Here we focus mainly on the most common types of single-cell DNA methylation sequencing technologies based on either restriction digestion (including methylation-insensitive and methylation-sensitive restriction endonucleases) or post-bisulfite adapter tagging (PBAT) (**Figure 2**).

### Restriction digestion-based DNA methylation profiling methods

Methods in this category rely on restriction endonucleases that recognize and cleave double-stranded DNA at specific sites. Combined with DNA size selection, these techniques allow analysis of the methylation status of targeted CpG sites with reduced sequencing costs. Assays based on methylation-insensitive endonucleases typically require the treatment of adapter-equipped DNA with sodium bisulfite, which converts unmethylated cytosine (C) to uracil and leaves methylated C unchanged. Therefore, unmethylated and methylated Cs can be accurately inferred from sequencing analysis 43. Conversely, the methylation-sensitive methods bypass the sodium bisulfite treatment. It only profiles unmethylated CpGs at cleavage sites, while the corresponding CpGs missed in the sequencing data are inferred as methylated 53. The characteristics of the two types of methods are summarized in **Table 2**.

#### Single-cell methylation sequencing method based on methylation-insensitive restriction enzyme

RRBS is the first NGS-based method for DNA methylation profiling 54. It relies on methylation-insensitive endonucleases, such as MspI (C|CGG), and size selection to cleave and enrich CpG-dense DNA fragments 55, 56. Several groups have published modified RRBS protocols by streamlining library processing, barcoding library DNA fragments to remove duplicates, or reducing genomic DNA inputs from microgram to picogram (single cell) levels 57-59. For example, Q-RRBS introduces 6-bp barcodes to the 5'- and 3'-ends of library DNA inserts, guaranteeing 4,096 adapter combinations and thus attempting to eliminate PCR-related duplicates 58. Microfluidic diffusion-based RRBS (MID-RRBS) uses a microfluidic device that allows DNA bisulfite treatment and subsequent purification steps to be performed in tiny chambers (240 or 480 nl). The modification minimizes DNA loss and allows profiling of DNA methylation changes with nanograms of DNA input or even with DNA from single cells 60. However, the method only captures about 35-231K CpGs in the mouse genome.

**Figure 3:**
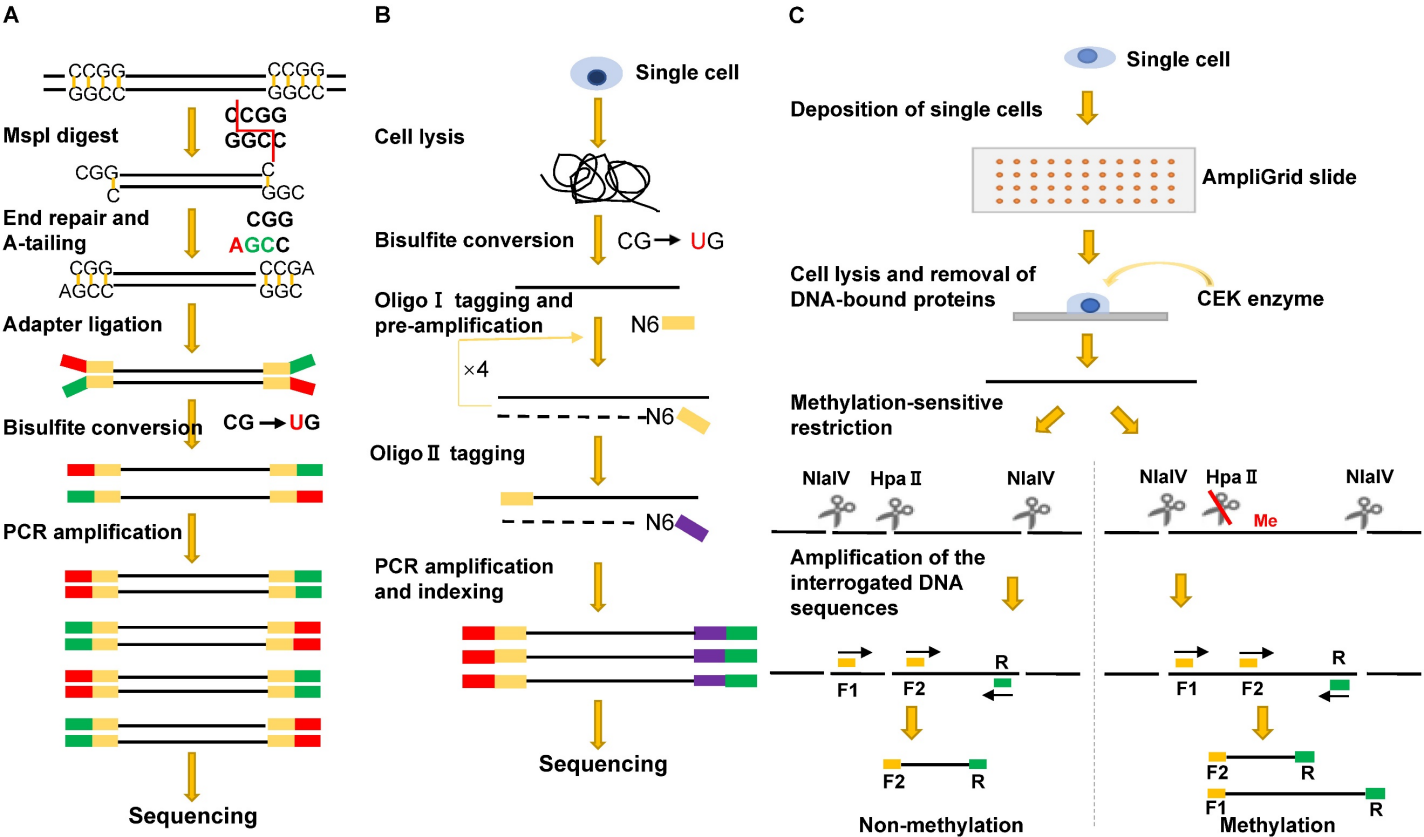
Schematic comparison of single-cell DNA methylation profiling methods: sc-RRBS (A), scBS-seq (B), and RSMA (C).

To generate single-cell RRBS (scRRBS) libraries, Guo and colleagues minimized the library DNA loss by performing five consecutive reactions from cell lysis, MspI digestion, end-repair, A-tailing to adapter ligation and the bisulfite conversion in one tube (**Figure 3A**). After two rounds of PCR enrichment, the amplified scRRBS libraries were pair-ended sequenced and the sequencing data indicated that scRRBS was capable of covering up to 1.5 million CpGs 57, 61. Using scRRBS to profile mouse sperm, oocytes, and zygotes reveals fine demethylation landscapes after fertilization 57. However, scRRBS can only process a limited number of single cells manually. The multiplexed single-cell RRBS (MscRRBS) is performed in a 96- or 384-well PCR plate and can be processed automatically 59, 62. By prefixing each cell with an inline barcode, dozens of adapter-equipped libraries can be pooled, dramatically reducing the subsequent workload and archiving coverage of up to 2 million unique CpGs for single human cells 62.

Extended-representation bisulfite sequencing (XRBS) deliberately uses Illumina adapters with unphosphorylated bottom strands. After the sodium bisulfite treatment, the converted DNA fragments have only a 5'-terminal adapter, and the 3'-terminal adapter sequences are introduced using random hexamer-tagged PCR primers. As a result, XRBS captures more CpG sites within two MspI cleavage sites 63. The single-cell XRBS (scXRBS) also barcodes each DNA sample prior to bisulfite conversion and PCR amplification. The modifications allow each scXRBS library to cover up to 3.43 million CpGs with less than 2 million reads and can identify PCR duplicates 63.

#### Single-cell methylation sequencing method based on methylation-sensitive restriction enzymes (MSREs)

MSREs are a group of restriction endonucleases that cannot cleave DNA if their recognition sites contain methylated cytosines. Interestingly, some have isoschizomers with identical recognition sequences but are insensitive to methylation. MSRE-based assays can use multiple enzymes to extend genomic coverage, and the missed CpG sites in the enzyme binding sites are methylated and inferred from the sequencing analysis 64. In contrast, Methyl-seq, which uses paired methylation-sensitive and methylation-insensitive isoschizomers MspI and HapII, can directly identify the methylation status of CpGs in their binding sites 65; however, this strategy is unsuitable for single-cell sequencing.

The first MSRE-based single-cell DNA methylation assay was described in 2011, and the method, restriction enzyme-based single-cell methylation assay (RSMA) (**Figure 3C**), can only detect a limited number of CpGs 66. The sequential reactions, including single-cell lysis, methylation-sensitive restriction digestion, and PCR amplification, are all performed on an AmpliGrid slide containing 48 microreactors for water-in-oil emulsions. The enzyme cleavage sites are located between the two forward primers so that the CpG methylation of the cleavage sites can be inferred either from the size of the PCR products or by pyrosequencing of the PCR product pool 66. Subsequently, Cheow et al. developed another MSRE-based single-cell methylation method called single-cell restriction analysis of methylation (SCRAM) by combining MSRE digestion and multiplex PCR amplification 67. The method applies a microfluidic qPCR chip and can detect the DNA methylation levels of 24 loci in up to 48 cells per assay. SCRAM is cost-effective but detects far fewer CpG sites than NGS-based single-cell DNA methylation assays. The method cannot distinguish between heterozygous methylated alleles and homozygous methylated alleles either. The single-cell CpG island methylation assay (scCGI-seq) is based on one round of MSRE digestion followed by multiplexed displacement amplification (MDA) and a second round of MSRE digestion 68. The method enables genome-wide measurement of CGI methylation levels from single cells (covering 76% of CGIs in the human genome). Although the coverage of CpG sites is lower than that of scWGBS, scCGI-seq shows good reproducibility across multiple single cells.

Single-cell targeted analysis of the methylome (scTAM-seq) is another MSRE-based sequencing technology 69. It can detect 650 CpG sites in up to 10,000 cells simultaneously. The assay uses a commercial microfluidic droplet device, the Mission Bio Tapestri platform, to mix individual cell lysate with barcoded beads tagged to gene-specific primers. Following methylation-sensitive restriction digestion and targeted PCR application in a thermal cycler, only targeted and methylated CpGs within the enzyme binding sites are amplified and sequenced. The application of scTAM-seq reveals the dynamic methylation status during B-cell differentiation in peripheral blood and bone marrow 69. Despite low coverage, scTAM-seq achieves an excellent high throughput and low false-positive rates of less than 0.2% 49, 69.

In contrast to MSRE-based assays, which generally detect symmetric DNA methylation on both the plus and minus strands, single-cell MspJⅠ-dependent sequencing (scMspJI-seq) is designed to assess strand-specific 5mC 70. The modification-dependent endonuclease MspJI targets mCNNR sites and cleaves downstream genomic DNA at approximately 9-13 bp. After the incorporation of double-stranded adapters containing T7 promoter, Illumina adapter, and unique molecular identifier sequences, DNA libraries are generated by *in vitro* transcription and PCR application of transcribed RNAs. Thus, scMspJI-seq specifically enriches methylated sites and has been used to study the dynamics of DNA demethylation in early development 70.

### PBAT-based single-cell WGBS

In conventional bisulfite-based sequencing methods, fragmented DNA is typically tagged by methylated adapters prior to bisulfite conversion. PBAT implements an initial bisulfite treatment protocol and then uses random primers to amplify bisulfite-converted DNA fragments, allowing more DNA fragments to be subsequently amplified and sequenced 71. Single-cell WGBS methods based on PBAT are summarized in **Table 3**.

Single-cell bisulfite sequencing (scBS-seq) is the first PBAT-based genome-wide methylation sequencing method in which bisulfite-treated DNA is subjected to two cycles of random primer extension 72, 73 (**Figure 3B**). Two critical steps, direct bisulfite treatment of the single-cell lysate and amplification of converted DNA before the purification of synthesized first-strand DNA, minimize DNA loss. In addition, the use of modified random hexamers eliminates the need to trim artificial bases introduced during conventional library preparation. On average, scBS-seq can detect 3.4 million CpGs per single cell. However, the method often fails to detect methylation differences for some alleles due to allele dropout caused by bisulfite conversion and enrichment-induced bias 49, 72.

The scWGBS method developed in the Bock laboratory primarily uses a commercial product, the EpiGnome™ Methyl-Seq Kit (Epicenter, EGMK81312), to generate sequencing libraries 74. Bisulfite-converted genomic DNA is first transcribed using tagged random hexamer primers, and then the 3′-terminal ends of the newly synthesized DNA strands are linked to a second specific sequence tag. scWGBS does not undergo pre-amplification, reducing reagent costs, processing time, and amplification bias 74. However, excessive PCR amplification to introduce Illumina-compatible sequencing adapters and generate library DNA negatively impacts library complexity, resulting in a relatively low coverage of approximately 1.4 million CpGs per cell.

Single-cell PBAT (scPBAT) uses a pair of primers, the Bio-PEA2-W4N4 primer (5'-biotin-ACACTCTTTCCCTACACGACGCTCTTCCGATCTWWWWNNNN-3') and the PE-index-W4N4 primer (5'-CAAGCAGAAGACGGCATACGAGATXXXXXXXXXGTAAAACGACGGCCAGCAGGAAACAGCTATGACWWWWNNNN-3') for the first- and second-strand DNA synthesis sequentially. It is noteworthy that scPBAT does not undergo PCR-based amplification and is tailored for methylation analysis of repetitive regions 75.

Some single-cell methylation libraries start from single-cell lysates, while others utilize single-cell nuclei instead, such as single-cell combinatorial indexing for methylation analysis (sci-MET), sciMETv2 linear amplification (sciMETv2.LA), sciMETv2 splint ligation (sciMETv2.SL), and single-nucleus methylcytosine sequencing (snmC-seq) 76-79. In the sci-MET assay, each nucleus is indexed by transposase tagmentation in a 96-well plate prior to pooling for bisulfite treatment, linear amplification of the bisulfite-converted DNA, and sequential PCR enrichment of the library pools. The sci-MET covers a low percentage of CpGs per cell (0.05-7.0%), but is capable of sequencing DNA methylation for thousands of cells and achieving high alignment rates of 60-76% 76. The optimized versions of sci-MET, sciMETv2.LA and sciMETv2.SL, achieve better tagmentation efficiency and increased coverage per cell, averaging 2.2 million and 325K unique CpGs, respectively 77. The improvement benefits from using methylated indexed tagmentation adapters and updated nucleosome disruption technology. The two sciMETv2 methods can identify cell subtypes in the human brain.

Both snmC-seq and snmC-seq2 rely on barcoded random primers to amplify bisulfite-converted DNA and on the adaptase (Swift Biosciences) to tag a short oligo tail at the 3'-terminal of synthesized DNA. Sequencing libraries are generated by PCR using a pair of custom indexing primers containing Illumina P7 and P5 sequences, respectively. Several modifications, including the use of a different degenerate random primer (RP-H9, H=A, T, C) and the deactivation of free random primers and dNTP, dramatically improve the library qualities of snmC-seq2 compared to snmC-seq, such as better mapping rates (64.7±2.6% vs. 52.4±4% for the mouse genome), fewer artifactual reads (6.1±5.2%) and improved library complexity (30.8±7.5% vs. 22.2±5.7) 79.

## Single-cell multi-omics sequencing

The emergence of single-cell genomic, epigenomic, and transcriptomic sequencing methods motivates scientists to explore technologies for parallel single-cell multi-omics profiling. Remarkably, single-cell multi-omics sequencing technologies have been reported and are summarized (**Table 4**). Applications of these technologies have greatly improved our understanding of cellular and molecular heterogeneities and the internal correlations within multi-omics in development and human disease.

Single-cell transcriptome and methylome sequencing allow the simultaneous assessment of gene expression and DNA methylation variation and the investigation of their correlation. Methodologically, most single-cell transcriptome and methylome sequencing assays have been developed by combining two types of single-cell sequencing methods. For example, Smart-RRBS combines Smart-seq2 and Msc-RRBS, scMT-seq is derived from scRRBS and Smart-seq2, and scM&T-seq is based on Smart-seq2 and scBS-seq 80-82. Single-cell triple omics sequencing (scTrio-seq) combines scRRBS and scRNA-seq, and the third layer of omics, copy number variation (CNV), is deduced from the scRRBS data 83.

The critical step in parallel RNA and DNA methylation sequencing is isolating DNA and mRNA from the same cell properly. Two methods are commonly used for this purpose. One is to completely lyse single-cells and then separate mRNA from DNA using oligo-dT-coated magnetic beads. The second method is to gently lyse the cell membrane to release the cytoplasm and mRNAs, then transfer the cytoplasm and mRNAs to a separate tube, leaving the genomic DNA in the nucleus for further processing. Both scM&T-seq and Smart-RRBS take advantage of oligo-dT-coated magnetic beads for DNA and RNA separation, while scMT-seq and scTrio-seq benefit from the separation of intact nuclei and the cytoplasm for subsequent processing 80-82, 84.

Nucleosome-free regions (NFRs) or accessible chromatin regions often overlap with transcriptional regulatory elements. Methods capable of simultaneously assessing the chromosomal accessibility and DNA methylation include single-cell nucleosome occupancy and methylation (scNOMe-seq) 85, single-cell chromatin overall omic-scale landscape sequencing (scCOOL-seq) 86, and improved scCOOL-seq (iscCOOL-seq) 87. scNOMe-seq relies on the GpC methyltransferase*,* M.CviPl, to catalyze the cytosine methylation of GpCs in NFRs. After bisulfite conversion of the M.CviPl-treated DNA and sequencing analysis, NFRs and endogenous DNA methylation are inferred according to the methylation patterns of regular CpGs and naturally unmethylated cytosines at GpC sites 85. The method is also developed from the bulk type NOMe-seq 88. By spiking in a certain amount of lambda DNA as an internal control, scCOOL-seq allows the profiling of individual cell ploidy 86. In addition, iscCOOL-seq offers a better mapping rate, 74.55% vs. 22.01%, compared to scCOOL-seq - the improvement benefits from the optimized protocol for constructing single-cell PBAT-based methylation libraries 87.

The single-cell multiple omics assay for genotype, gene expression, and methylation profiling (sc-GEM) combines the single-cell restriction analysis of methylation (SCARM) technique with NGS-based single-cell genotyping. Targeted-gene transcripts are assessed by qPCR. Most of the experimental procedures are performed on the Fluidigm C1 single-cell auto-prep system 89, 90. In particular, the methylation analysis is based on the digestion of an MSRE, HpaII (5'-C|CGG-3'), followed by qPCR amplification on the Fluidigm array. The assay covers a limited number of genes and gene transcripts; however, by performing the test on the Fluidigm instrument, hundreds of single cells can be analyzed simultaneously 89. Another assay that can measure DNA methylation and identify genetic variants is epi-gSCAR (epigenomics and genomics of single cells analyzed by restriction) 91. The method is based on Hhal, an MSRE that recognizes 5'-GCG|C-3' and can significantly enrich for CGIs and transcription start sites (TSSs). Sequencing analysis of epi-gSCAR libraries can detect up to half a million CpG sites and 1.2 million single-nucleotide variants (SNVs) 91.

Single-cell nucleosome, methylation, and transcription sequencing (scNMT-seq) can concurrently evaluate chromatin accessibility, DNA methylation, and gene transcription by applying M.CviPI to label the open chromatin regions. The method also uses oligo-dT-coated magnetic beads to precipitate mRNAs for RNA-seq library construction, leaving M.CviPI-treated DNA in the lysate for methylation analysis 92. scNMT-seq can detect methylation changes in approximately half of the mouse promoters, three-quarters of gene bodies, and one-quarter of enhancers. Similarly, scNOMeRe-seq integrates scNOMe-seq and multiple annealing and dC-tailing-based quantitative single-cell RNA sequencing (MATQ-seq) to profile chromatin accessibility, DNA methylation, and gene transcription of the same cell 93, 94. Unlike scNMT-seq, in which single cells are FACS sorted, scNOMeRe-seq is based on manually picking single cells and transferring the cytoplasm to another tube for MATQ-seq, leaving the nuclei for the GpC methylase treatment followed by scBS-seq 77. scNOMeRe-seq can detect 3.49 million CpGs per single cell and more than 1000 gene transcripts for 94.8% of single cells 78.

## Biological applications of single-cell DNA methylation sequencing

**Figure 4:**
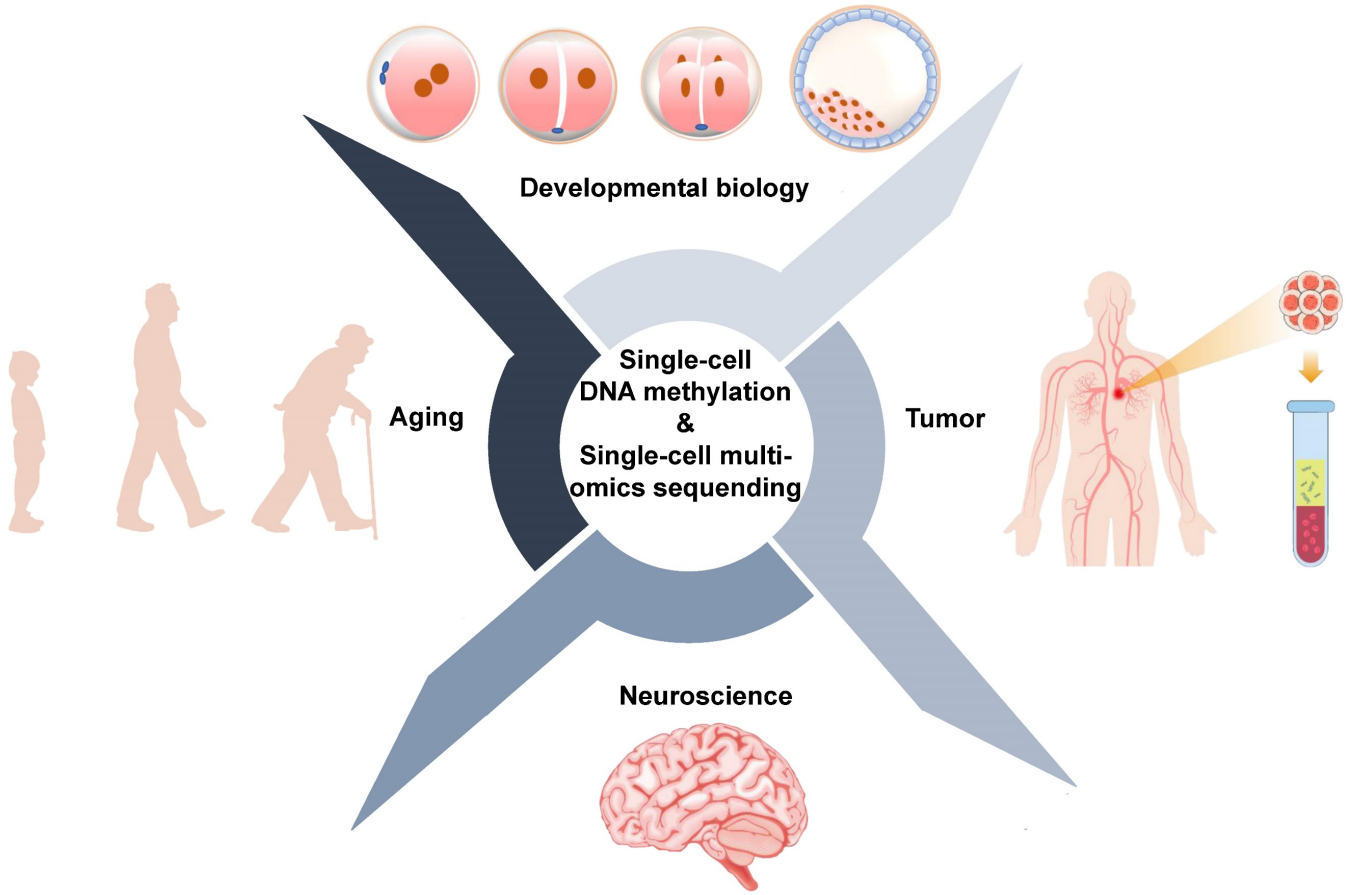
Biological applications of single-cell DNA methylation sequencing and single-cell multi-omics sequencing.

Conventional sequencing approaches require thousands to millions of cells and provide average changes at the genetic, epigenetic, and transcriptional levels. However, bulk sequencing technologies cannot reveal what is happening in rare cells or subpopulations of cells. Single-cell sequencing technologies provide tools to precisely profile DNA methylation and other omics for individual cells. Applications of single-cell DNA methylation and single-cell multi-omics sequencing are primarily focused on the development and human disease, particularly cancer 95 (**Figure 4, Table 5**).

### Application of single-cell DNA methylation sequencing in developmental biology

Mammalian life begins at fertilization, where both paternal and maternal genomes undergo global demethylation, reaching its lowest level at the blastocyst stage 14, 96. Using single-cell PBAT-based WGBS, Zhu and colleagues showed that local remethylation is interspersed with global demethylation. The authors further showed that methylation levels decrease more rapidly in the paternal genome, resulting in the paternal genome having consistently lower methylation levels from the two-cell stage to the blastocyst stage 97. The same group further profiled DNA methylation and chromosome accessibility of early human embryos using scCOOL-seq. The results indicate that the chromatin of the paternal genome tends to be more open compared to the maternal genome shortly after fertilization up to the 4-cell stage 98.

Mouse is the most commonly used model animal to study early mammalian development. Single-cell DNA methylation analysis of the paternal and maternal genomes in mouse zygotes shows that the demethylation process of the genic region is faster than that of the intergenic regions 57. Simultaneous profiling of the methylome and transcriptome of mouse embryonic stem cells by scM&T reveals novel correlations between the methylation patterns of regulatory elements and the expression of pluripotent genes 81. Using scNOMeRe-seq, Wang et al. mapped the chromatin accessibility, detected DNA methylome variation, and profiled the transcriptomes of the mouse preimplantation embryos at the single-cell level. The authors also constructed genetic lineages from zygotes to the 8-cell stage and demonstrated that asymmetric cleavage may result from the transcriptional heterogeneity of blastomeres 93.

After blastocyst implantation, DNMT3A and 3B catalyze *de novo* methylation of the genome 14, 96. Single-cell triple omics sequencing reveals that the genome remethylation of the primitive endoderm (PrE) cells is slower than that of the epiblast and trophectoderm cells, despite the fact that PrE and epiblast are both derived from the inner cell mass 99.

### Application of single-cell DNA methylation sequencing analysis in tumors

Extensive studies show that epigenetic abnormalities are closely associated with the development and evolution of cancer 100-102. Genome-wide hypomethylation and focal hypermethylation, particularly at the promoters of tumor suppressor gene, have been implicated as hallmarks of cancer 103-105. Although observations based on 'bulk' DNA methylation analysis are likely valid, the superiority of single-cell sequencing analysis for cancer studies is evident. First, solid tumor tissues contain many cell types, including cancer cells, fibroblasts, endothelial cells, and infiltrating immune cells and nerves 106. Therefore, bulk sequencing may not faithfully reflect the genetic and epigenetic status of tumor cells. Second, different subclones may coexist within the same tumor, and epigenetic plasticity permits cancer cells to alter their cellular state in response to microenvironmental and therapeutic stimuli 107. Both directly contribute to the complexity of tumor heterogeneity. Finally, the accessible tumor cells may be limited, such as circulating tumor cells (CTCs) in the peripheral blood of cancer patients.

Cellular heterogeneity is closely associated with cancer development, evolution, and response to treatment. Many studies have used single-cell DNA methylation sequencing to investigate cellular heterogeneity in cancers, such as colorectal cancer, breast cancer, liver cancer, and chronic lymphocytic lymphoma (CLL) 59, 83, 108-110. One study evaluates genetic, epigenetic, and transcriptional abnormalities in colorectal cancer using scTric-seq2 to analyze single cells derived from primary, lymphatic, and metastatic tissues 111. The study identifies significant differences in overall methylation levels between genetic sublineages but less variation within a sublineage. Interestingly, the demethylation patterns of cancer cells are comparable across all ten patients 111. An independent study investigates 914 single-cell methylomes, 55,284 single-cell transcriptomes, and bulk multi-omics sequences from 11 glioma patients with or without isocitrate dehydrogenase (IDH) gene mutation 112. The study suggests that aberrant methylation is associated with early genetic alterations and that accumulated genetic alterations are related to altered cellular states and environmental stresses.

Understanding tumor heterogeneities and clonal evolutionary trajectories could help scientists elucidate the underlying mechanisms and develop specific targeted drugs. Using scTrio-seq, Hou et al. reported two subpopulations based on the CNV, methylation, and transcriptional profiles of 25 single cells isolated from the liver tissue of one patient with hepatocellular carcinoma 83. The authors also found cellular heterogeneity within the subpopulations. Single-cell sequencing analysis not only sheds new light on solid tumor research but also provides mechanistic insight into chronic lymphocytic lymphoma (CLL). By applying Msc-RRBS to B cells from CLL patients and healthy donors, Gaiti and colleagues constructed the lineage tree and showed different branching patterns and lengths in the two cell populations 59. Further analysis of the B cells using Smart-RRBS identified an ibrutinib-related bias in the methylation-based lineage tree, demonstrating how the therapeutic intervention affects the clonal evolutionary trajectory of CLL patients. Moreover, the upregulation of multiple Toll-like receptor (TLR) signalling pathway genes in ibrutinib-treated patients suggests a new direction for the development of targeted therapy 59.

Single-cell multi-omics sequencing technology is able to identify differentially expressed and differentially methylated genes in colorectal cancer, which can be used as biomarkers to guide targeted therapy for patients 113. In one single-cell multi-omics study, DNA methylation is linked to the clonal stability of colorectal cancer cells and is strongly associated with cancer progression 114. By simultaneously profiling the methylome, chromatin accessibility, and transcriptome, Fan et al. showed that hypermethylation is common in heterochromatin regions in the genome of patients with pancreatic ductal adenocarcinoma. In contrast, hypomethylation is typical in euchromatin regions. The authors also identified two biomarkers, ZNF667 and ZNF667-AS1, and showed that expression of these biomarkers is associated with a better prognosis 115.

Circulating tumor cells (CTCs) are cancer cells shed from primary or metastatic tumors into the peripheral blood. CTCs are rare, and often fewer than ten cells can be isolated from 10 ml of peripheral blood 116. However, CTCs carry intact genetic, epigenetic, and transcriptional characteristics of tumor cells, making them ideal for studying tumor biology and monitoring tumor development and evolution. It is, therefore, possible to trace the cancer tissue of origin. The hypothesis was tested by applying scBS-seq to CTCs from six cancer types, and the investigation revealed tumor heterogeneities and an evolutionary pathway during cancer metastasis. The tumor tissue origin was also successfully identified based on the methylation landscapes of CTCs 117. In addition, a systemic evaluation of the DNA methylation patterns of single CTCs and clustered CTCs reveals hypomethylation of binding sites for stemness- and proliferation-associated transcription factors (TFs), particularly in clustered cells 118. The study demonstrates that an FDA-approved compound, a Na+/K+ ATPase inhibitor, disrupts CTC clustering, alters DNA methylation at TF-binding sites, and inhibits metastasis 118. Another research using targeted bisulfite sequencing for three-EMT (epithelial-to-mesenchymal transition) genes tested 159 single CTCs from breast or prostate cancer patients. The study concluded that the methylation profiles of CTCs mirror those of epithelial-like cells and that CTCs have different methylation levels 119.

### Single-cell DNA methylation sequencing in neuroscience and aging

Applications of single-cell DNA methylation sequencing and single-cell multi-omics sequencing technologies have also been extended to other research areas, such as neuroscience and aging. DNA methylation in neurons exhibits a unique feature, with a significant amount of methylated cytosine at CpH sites (H=A/T/C) in post-mitotic human and mouse neurons 120-122. Notably, both CpG and non-CpG methylation are essential for neuronal development in the brain 120, 121, 123. Single-cell methylation analysis of >6000 mouse and human frontal cortex neurons classifies these cells into 16 mouse and 21 human subpopulations, and both CpG and non-CpG methylation show cell-type-oriented landscapes 78. In a parallel study, Liu and coworkers generated a brain DNA methylation atlas using 103,982 nuclei from 45 mouse brain regions. Single-cell methylation analysis reveals 161 subpopulations with distinct spatial locations and projection targets 124. The integration of single-cell DNA methylation and chromatin accessibility datasets ultimately provides an epigenetic atlas for interpreting gene-enhancer interactions and understanding the 3D structure of neurons throughout the mouse cerebrum 124, 125.

A hallmark change of aging is genome-wide DNA hypomethylation 126. Accordingly, DNA methylation-based biomarkers have been evaluated for predicting age and are considered the most promising of six distinct age estimators 127. Recently developed pan-tissue epigenetic clocks can accurately estimate age using virtually any tissue from any mammalian species, suggesting that highly conserved DNA methylation patterns exist across mammals 128. Gaiti and collaborators created a molecular clock based on the single-cell methylation dataset of a CLL patient. The authors predicted the subclonal divergence in the evaluation path and showed that the ancestral clone had evolved 2,180 ± 219 days, suggesting that the molecular clock could guide the treatment of CLL patients 59. Another hallmark change is increased epigenetic or transcriptional heterogeneity during aging 129. However, conventional bulk sequencing assays are unable to detect cell-to-cell variability. One study exploits the joint profiling of the single-cell transcriptome and single-cell methylome of mouse muscle stem cells. The assay reveals aged stem cells with increased transcriptional heterogeneity and localized DNA methylation changes, suggesting epigenetic drafting during aging 130. Likewise, single-cell DNA methylation analysis of young and old mouse livers shows that mouse liver DNA methylation levels are highly variable, with an epivariation rate of 3.3%. Furthermore, DNA methylation heterogeneity is associated with genomic characteristics 131.

## Perspectives

Over the last two decades, DNA methylation profiling technologies have changed dramatically from Sanger sequencing-based low-throughput to NGS-based high-throughput, from bulk DNA/RNA inputs to requiring only single cells 47. Many single-cell DNA methylation sequencing technologies are currently available with varying coverage and mapping rates. However, improved CpG coverage often comes at the cost of reduced reproducibility. Different sequencing technologies can jointly provide comprehensive and accurate interpretations of genetic, epigenetic, and transcriptional changes. As illustrated earlier, most single-cell DNA methylation sequencing methods are based on bisulfite treatment, which causes significant DNA degradation and limits library complexity 132, 133.

Conversely, TET-assisted pyridine borane sequencing (TAPS) is based on TET oxidation of 5mC and 5hmC to 5caC, followed by pyridine borane reduction of 5caC to dihydrouracil. Enzyme-based bisulfite conversion is milder and generally does not cause DNA damage. TAPS can effectively identify modified cytosines with better mapping rates and uniform coverage 134. However, the method requires a large amount of DNA input, and TAPS-based single-cell assays are not yet available as we draft the manuscript. In addition, most single-cell DNA methylation methods cannot process large numbers of cells, although many of them have improved throughput, such as sci-MET and Smart-RRBS 76, 80. The development of efficient and high throughput assays is needed to analyze millions of CpG sites in hundreds or even thousands of single cells at a time in the future.

Sequencing costs have fallen dramatically over the last two decades, but profiling genetic and epigenetic changes at the single-cell level remains a challenge for many academic laboratories. One critical reason is that single-cell-based assays typically require sequencing hundreds or even thousands of single cells to obtain a comprehensive population-level picture 59, 112, 135. A prototype sequencer from Ultima Genomics (Ultima), which adopts the mostly natural sequencing-by-synthesis (mnSBS) chemistry, can sequence the human genome with sufficient coverage at a cost of $100 136, 137. The new sequencer significantly reduces the sequencing cost and sheds new light on single-cell sequencing. However, whether it can be used to profile the methylome requires further investigation.

Single-cell DNA methylation sequencing has been widely used to profile rare cells and investigate cellular heterogeneity. CTCs preserve tumor genetic and epigenetic information well and are excellent candidates for cancer prognosis and diagnosis 138-140. It is foreseeable that single-cell methylation sequencing and site-specific methylation assays will be incorporated into clinical testing. In addition to cancer, many publications report aberrant DNA methylation in other diseases, such as cardiovascular disease (CVD) 141, 142. One study investigates whether the prevalence of CVD is associated with the global genomic DNA methylation levels in peripheral blood leukocytes (PBL) in a cohort of 286 Singaporean Chinese 143. The study shows that increased DNA methylation is positively associated with the prevalence of CVD. In a recent case-control study involving thousands of participants, Fernandez-Sanles et al. identified 34 CpGs associated with acute myocardial infarction and four strongly correlated with coronary heart disease (CHD) and CVD 144. However, how DNA methylation contributes to the development of CVD is still not fully understood 141. The above single-cell-based assays will provide tools to dissect the molecular mechanism of CVD and identify biomarkers for diagnosis and prognosis of the disease.

Finally, the role of DNA methylation in gene regulation is complex 5, 23, 145. For example, increased DNA methylation at promoter regions is generally thought to be anti-correlated with gene expression 17, 54, 146. The relationships between gene expression and gene body methylation appear to be cell type dependent, being positively correlated in embryonic stem cells and negatively correlated in neurons 8, 14, 121, 147. Furthermore, single-cell multi-omics sequencing shows that only a small percentage of promoter methylation levels are negatively associated with gene expression 81, 92, 148. Similarly, significant correlations are only observed for a few gene bodies 81, 82. The application of single-cell DNA methylation and single-cell multi-omics sequencing technologies across different cell types will help to elucidate the precise function of DNA in gene regulation in the coming years.

## Figures and Tables

**Table 1:** Advantages and disadvantages of single-cell isolation methods

Isolation methods	Advantages	Disadvantages
Random limiting dilution	Easy to operate, no special equipment required	Less efficient
Mouth pipetting	Low cost and virtually no cell damage	Difficult to operate
Micromanipulation	Cost-effective and accurate single cell access	Difficult to operate
Laser microdissection	Retention of spatiotemporal information	Low throughput
Microfluidic devices	High-throughput cell sorting based on cell surface markers	Expensive consumables
Flow cytometry	High throughput, cell sorting using antibodies and fluorescent markers	Requires large number of cells, high cost, harmful to cells

**Table 2:** Single-cell DNA methylation profiling method based on restriction endonuclease digestion

Method	Strategies	Coverage per cell*	Throughput	Advantages	Disadvantages	Applications	Ref
scRRBS	One-tube enzymatic reactions	∼1.0M CpGs (mouse, mean coverage)	10s of cells	High promoter/CGI coverage	Sequence bias due to two rounds of PCR application	DNAm dynamics in development and disease	57
Q-RRBS	Unique molecular identifiers	0.5-1M CpGs (human)	1-100 cells	Eliminate PCR-derived duplication; detect allele-specific methylation	Low CpGs coverage	DNAm dynamics in development and disease	58
MscRRBS	Inline barcode, one-well enzymatic reactions	∼0.9M CpGs (human, mean coverage)	384 cells	High-throughput and easy set-up with automation	Low CpGs coverage	DNAm dynamics in development and disease	62
scXRBS	Unphosphorylated adapter, a random hexamer to add the second adapter	up to 3.4M CpGs (human)	96 cells	Extended CpG coverage for regulatory elements	Complicated library preparation	CNV and DNAm changes across single cells	63
MID-RRBS	Reactions in a microfluidic device	35k-231K CpGs (mouse)	96 cells	Efficient bisulfite conversion with increased DNA recovery	Low CpG coverage for single cells; requires non-commercial instrumentation	Cell type-specific epigenetic drug screening and drug-methylome interaction studies	60
RSMA	Reactions in a multi-well PCR slide	10-20 CpGs (human)	48 cells	Cost-effective	Unable to confirm heterozygous methylation	CpG methylation status in many single cells	66
SCRAM	PCR using two forward primers and one reverse primer	up to 24 CpGs (mouse)	48 cells	High reliability and accuracy	Not suitable for genome-wide screening DNAm	Targeted CpG methylation changes	67
scCGI-seq	Two rounds of MSRE digestion, MDA	∼21K CpGIs (human)	10-100 cells	High and consistent CpGI coverage	Low CpGs coverage, low-throughput	DNAm heterogeneity in development, differentiation and cancer	68
scMspJI-seq	Enrich methylated CpGs with MspJI	212-977K CpGs (mouse)	384 cells	Cost-effective	Difficult to map allele-specific DNA methylation	Strand-specific DNAm; investigation of mechanisms regulating demethylation dynamics	70
scTAM-seq	Tapestri platform; gene-specific primers	650 CpGs (human)	up to 10k cells	High-throughput; automation	Low CpG coverage	Targeted CpG methylation changes in development and disease	69

Notes: *Description in parentheses indicates sample source for assay development. DNAm--DNA methylation; MDA--Multiple Displacement Amplification.

**Table 3:** Single-cell DNA methylation profiling methods based on PBAT

Method	Strategies	Coverage per cell*	Throughput	Advantages	Disadvantages	Applications	Ref
scBS-seq	Two rounds of random priming	∼3.7 M CpGs (mouse, mean coverage)	96 cells	Genome-wide CpG coverage	Difficult to detect allele-specific methylation	DNAme in rare cells and heterogeneous populations	72
scWGBS	Integration of sequencing adapters using tagged random hexamers and terminal tagging	∼1.0 M CpGs (mouse, mean coverage)	96 cells	Fast, cost-effective	Low library complexity	DNAm dynamics in development and disease	74
scPBAT	Targeting DNAm at repetitive elements	0.7%-6.6% (mouse)	1-100 cells	Low sequencing costs and low PCR bias	Low mappability ofsequencing reads	Role of intergenic DNAm in mammals and other vertebrates	75
snmC-seq	Adapter sequence incorporation using random primers and adaptase	22.2 ± 5.7% (mouse)	384 cells	Improved mappability	High levels of adapter dimer sequences	Role of DNAm in disease, drug screening and cognition	78
snmC-seq2	Adapter sequence incorporation using degenerate random primers and adaptase	30.8 ± 7.5% (mouse)	384 cells	Increased throughput, reduced artificial reads and improved library complexity	Adapter dimer sequences of about 10%	Role of DNAm in disease, drug screening and cognition	79
sci-MET	Transposase tagmentation, linear amplification, indexed PCR enrichment	0.05-7% (human)	96 cells	High-throughput potential and improved mappability	Low coverage, custom sequencing recipe & primers	DNAm alterations in cancer and neurological disorders	76
sciMETv2.LA	Indexed tagmentation, streamlined linear amplification, and indexed PCR amplification	∼2.2 M CpGs (human, mean coverage)	96 cells	High-coverage	DNA libraries with short-inserts, read pairs not fully overlapped	DNAm alterations in cancer and neurological disorders	77
sciMETv2.SL	Indexed tagmentation, splint ligation to add the TruSeq I5 adapter, and indexed PCR amplification	∼0.3 M CpGs (human, mean coverage)	96 cells	Low cost and less processing time	Low-coverage	DNAm alterations in cancer and neurological disorders	77

Notes: *Description in parentheses indicates the sample source for assay development. DNAm--DNA methylation; MDA--Multiple displacement amplification.

**Table 4:** ** Summary of** single-cell multi-omics sequencing technologies

Name	Omics	Methodology	Ref
Smart-RRBS	Transcriptome, methylome	Smart-seq2, MscRRBS	80
scMT-seq		Smart-seq2, scRRBS	82
scM&T-seq		Smart-seq2, scBS-seq	81
scTrio-seq	Transcriptome, methylome, CNVs	Smart-seq, scRRBS	83
scNMT-seq	Transcriptome, methylome, chromatin accessibility	Smart-seq2, NOMe-seq	92
iscCOOL-seq		scCOOL-seq, NOMe-seq	87
scNOMeRe-seq		scNOMe-seq, MATQ-seq	93
scGEM	Methylome, transcriptome	SCRAM	89
epi-gSCAR	Methylome, genetic variants	MSRE	91
scNOMe-seq	Methylome, chromatin accessibility	NOMe-Seq	85
scCOOL-seq	Methylome, chromatin accessibility, ploidy	COOL-seq	86

**Table 5:** Biological applications of single-cell DNA methylation sequencing

Application	Method	Conclusion	Ref
Developmental biology	scBS-seq, Smart-seq2	Dynamic DNAm during preimplantation with global demethylation and localized remethylation.	97
	scCOOL-seq	Major changes in chromatin state and DNA methylation do not occur simultaneously after fertilization.	98
	scRRBS	Genic regions demethylated faster than intergenic regions in early mouse embryo development.	57
	scM&T-seq	Methylation patterns of distal regulatory regions correlate with gene expression.	81
	scNOMeRe-seq	DNAm remodeling is essential for reconstructing genetic lineages in early embryos.	93
	scTrio-seq2	Genome remethylation in primitive endoderm cells is slower than in epiblast and trophectoderm cells.	99
Tumor	scTrio-seq	Identification of cancer cell subpopulations and cellular heterogeneity within a subpopulation.	83
	scTrio-seq2	DNAm variation between primary and metastatic colorectal tumors reflects different sublineage composition.	111
	scRRBS	Abnormal DNAm in gliomas is associated with early genetic changes, and accumulated genetic variation is due to altered cellular states and environmental stress.	112
	Msc-RRBS, Smart-RRBS	Illustration of the lineage history of CLL and its evolution under pharmacological treatment.	59
	MARS-seq	Epigenetic memory diversifies the genetic subclonal structure of cancer cells.	114
	scCOOL-seq	Detection of enriched DNA demethylation in heterochromatin regions in pancreatic ductal adenocarcinoma (PDAC) and identification of two candidate biomarkers for the diagnosis of PDAC.	115
	scBS-seq	Classification of tumor origin using DNAm landscapes of CTCs.	117
	scWGBS	Hypomethylation of CTC clusters associated with poor prognosis in breast cancer.	118
Neuroscience	snmC-seq	Establishment of a comprehensive DNAm atlas of mammalian neurons, demonstration of the essential role of epigenetic diversity in neuronal development.	78
	snmC-seq2	Creation of a sophisticated DNAm atlas of the mouse brain.	124
Aging	sc-DNAm	DNAm as an epigenetic clock for age estimation in mammals.	128
	scM&T-seq	Aging is associated with a global increase in transcription and methylome heterogeneity.	130
